# Correspondence

**Published:** 1900-07

**Authors:** Leonard L. Conkey

**Affiliations:** Dean, Grand Rapids Veterinary College, Michigan


### CORRESPONDENCE.

GRAND RAPIDS, MICHIGAN, April 2, 1900.

EDITOR JOURNAL OF COMPARATIVE MEDICINE, ETC.

DEAR SIR AND EDITOR: As Mr. Jopling, who holds a certificate from the Ontario Veterinary School, and not unlike many others of his kind in Michigan, who are blessed with those foreign certificates, follows another vocation for a livelihood, has been elected President of the Michigan Veterinary Medical Association, in which official capacity he has seen fit to attack the Grand Rapids Veterinary College and its Dean, we trust that you will accord us space in the JOURNAL for a reply to his unique effusion.

Mr. Jopling (we use the term Mr. because the Ontario Veterinary School never was, and perhaps never will be, clothed with authority to confer the degree of Doctor upon any man) says that one of the many pitfalls that has confronted their committee on legislation is a certain garrulous individual hailing from Grand Rapids, etc.

Now, let us dissect this liveryman's assertion. The records of the House of Representatives of Michigan are proof of the following statements: That on February 16, 1893, a bill was introduced by the Hon. A. S. White, of our city, for the regulation of the practice of veterinary medicine. This bill was drafted by the undersigned, who is now the Dean of the Grand Rapids Veterinary College. A few days later a bill was introduced by the Hon. Mr. Hammond, who represented the Michigan State Veterinary Medical Association. The Hammond bill was framed with an eye single to the glory of the Ontario Veterinary School, consequently it met with opposition.

The Association's committee, of which the Hon. W. W. Thorburn, was chairman, invited us to meet with them in Lansing; at this meeting a truce was patched up by amending the Hammond bill, which was to be pushed by petitions, and the White bill was dropped. Returning home we circulated petitions in several counties, and alone secured more signers than did all the members of the Association combined—as to the truth of this W. W. Thorburn, then chairman of the committee, can testify. The Hammond bill was placed in the hands of an attorney, employed by the Association as lobbyist, and has never been heard from since. Therefore we ask, who was responsible for its downfall?

During the year 1895 the Association could not agree on any one measure, and while they were engaged in a war of words we made a second attempt to better the standing of our profession. Singling out Representative Chilver as our Moses, April 5, 1895, he introduced House Bill No. 500. At this time we again expended more than a hundred dollars travelling about the State soliciting aid in support of the measure. This bill passed the House with but one immaterial change, and it was the first veterinary bill that ever did pass the House.

It went to the Senate where it was met by the Association in the person of Dr. E. A. A. Grange, then State Veterinarian, and by the efforts of the Association through Grange the bill was tabled only a day or two before the final adjournment of the Senate.

Again we ask, who was responsible for its downfall?

During the year 1897 the Association was still sparring; the Americans arrayed on the one side and the foreign element on the other.

February 15, 1899, House Bill No. 18 was introduced by the Association. This bill passed the House before we saw it and went to the Senate. The Hon. R. T. Graham sent us a copy, on receipt of which we telegraphed the Senate to hold the bill until we could be heard; this request was granted.

We saw at a glance that the bill would legislate out of practice scores of competent veterinarians, who had fully complied with all the requirements of some of the leading veterinary colleges of the United States at the time they obtained their degree, and who have been active and reputable practitioners for many years.

The ring-leaders of the Association had displayed about as much sense as did the man who contracted to scuttle a ship in mid-ocean. The bill possessed but little merit, as it did not prevent unqualified persons from practising veterinary medicine; applying only to the professional title. We modestly, yet firmly insist that it was inferior to the bill prepared by competent attorneys at our request, and introduced by Representative Anderson, of our city, a copy of which is in your possession; we further state that any one of the foregoing measures has more merit than does the one that has become a law, gladly making you judge of them.

Notwithstanding all this we bent our efforts to have the association bill amended for its betterment, and believe that in the opinion of every veterinarian in the State, except the "foreign element," we succeeded. But the "foreign element" is disgruntled; first, because they find that "there are others" as our noble Governor Pingree told them, and second, because he (Governor Pingree) refused to be hoodwinked into appointing their slate to positions on the State Veterinary Board of Examiners, which Board they had hoped to control in the interest of the Ontario Veterinary School; hence the cries of "Quack," the "Grand Rapids Mill," etc.

Now, Mr. Editor, let us quote Webster's definition of "Quack," and if we come within his definition we shall cheerfully adopt that cognomen. Webster: "A quack is a boastful pretender to medical skill, hence one who boastfully pretends to knowledge not possessed; an ignorant, pretentious practitioner in any branch of knowledge."

With this as a definition we brand Mr. Jopling a malicious falsifier, as we never boasted of knowledge not possessed, and we will put up five hundred dollars against a like sum that we can to-day, or any day, pass an examination at a higher percentage in all branches of veterinary science, including clinical surgery, before the State Board of Examiners than can Mr. Jopling, President of the M. S. V. M. A. We will include in this challenge any member of their Association.

Now, Jopling, come up and take this examination. You can do it in one day, and five hundred dollars is more money than you can make in the livery business in six months.

"The Grand Rapids Mill." While we are ready to brand any man a liar that applies to our college this title, yet from a financial point of view it might be well to get a little advertisement in this line. We have heard no other name for the Ontario Veterinary School since we can remember; nevertheless there were one hundred and forty students at the Ontario Veterinary School during the last session. It appears that there are a whole lot of people that want a diploma, even though they do, like some of the officials of the M. S. V. M. A., come within Webster's definition of a "Quack" after they get it. However, that your readers may know the relation between our College and the State officials, we beg to append two clippings from the Lansing *Republican* and one from the Detroit *Free Prees.*

The *Secretary of the State Board of Veterinary Examiners* called on Attorney-General Oren yesterday and sought to have that official institute proceedings to put the Grand Rapids Veterinary College out of business. He was advised that his showing did not warrant any such action.

REPORT UNFOUNDED.—*Dr. W. W. Thorburn, Secretary of the State Board of Veterinary Examiners,* stated to-day that the report that the State Board was trying to put the Grand Rapids Veterinary College out of business was a misrepresentation of the views of the Board, given to the press by an outside party. The Board is not opposed to any college which comes up to the requirements of the law.—*Lansing Republican.*

OBJECTIONS HAVE BEEN REMOVED.—W. A. Giffen, of Detroit, Secretary of the State Veterinary Medical Association, has been here consulting with the attorney-general in regard to the Grand Rapids Veterinary College, the graduates of which, it is claimed, are not entitled to practise their profession under the new veterinary registration law. Mr. Giffen is understood to have obtained but little satisfaction, as the college in question has been examined by State authorities, whose objections to its methods have been removed.—*Detroit Free Press,* March 27.

Now, Mr. Editor, it will be seen by comparing the bills that the words "Citizens of the United States" have been inserted; also, the words in Sec. 3, line 1, "Not an alien." This was done at our suggestion, as we do not believe this State should, by legislation, favor the foreign element or foreign institutions by the enactment of un-American laws.

This was another severe blow to the "foreign element," and one that is being resented in a mean, underhanded way, and as we were directly responsible for those changes their vengeance is being wreaked on the Grand Rapids College.

Again, Mr. Editor, as to the length of our College course. We have consulted members of the State Veterinary Board and we are informed that some of the Board are opposed to the recognition of diplomas from any foreign school. Were we certain that the Board would adopt this policy we would extend our curriculum at once to three years, but inasmuch as the "foreign element" are active in their endeavor to change the complexion of the Board we cannot decide upon a change now.

In future, please do not trouble yourself about endowments or State assistance for our College, as the proceeds from our hospital and veterinary practice, aside from the College, are sufficient to meet all running expenses, and believing that education of every kind should be placed within the reach of as many as possible, we are seriously thinking of reducing the College fees to fifty dollars, including laboratory and anatomical fees.

Yours, with personal regards,

LEONARD L. CONKEY, Dean,
Grand Rapids Veterinary College.

